# Selection of Cyanobacterial (*Synechococcus* sp. Strain PCC 6301) RubisCO Variants with Improved Functional Properties That Confer Enhanced CO_2_-Dependent Growth of Rhodobacter capsulatus, a Photosynthetic Bacterium

**DOI:** 10.1128/mBio.01537-19

**Published:** 2019-07-23

**Authors:** Sriram Satagopan, Katherine A. Huening, F. Robert Tabita

**Affiliations:** a Department of Microbiology, The Ohio State University, Columbus, Ohio, USA; University of Georgia; Indiana University Bloomington; University of Georgia

**Keywords:** RubisCO, carbon dioxide fixation, directed evolution, enzyme engineering, selection

## Abstract

Ribulose 1,5-bisphosphate carboxylase/oxygenase (RubisCO) is a ubiquitous enzyme that catalyzes the conversion of atmospheric CO_2_ into organic carbon in primary producers. All naturally occurring RubisCOs have low catalytic turnover rates and are inhibited by oxygen. Evolutionary adaptations of the enzyme and its host organisms to changing atmospheric oxygen concentrations provide an impetus to artificially evolve RubisCO variants under unnatural selective conditions. A RubisCO deletion strain of the nonsulfur purple photosynthetic bacterium Rhodobacter capsulatus was previously used as a heterologous host for directed evolution and suppressor selection studies that led to the identification of a conserved hydrophobic region near the active site where amino acid substitutions selectively impacted the enzyme’s sensitivity to O_2_. In this study, structural alignments, mutagenesis, suppressor selection, and growth complementation with R. capsulatus under anoxic or oxygenic conditions were used to analyze the importance of semiconserved residues in this region of *Synechococcus* RubisCO. RubisCO mutant substitutions were identified that provided superior CO_2_-dependent growth capabilities relative to the wild-type enzyme. Kinetic analyses of the mutant enzymes indicated that enhanced growth performance was traceable to differential interactions of the enzymes with CO_2_ and O_2_. Effective residue substitutions also appeared to be localized to two other conserved hydrophobic regions of the holoenzyme. Structural comparisons and similarities indicated that regions identified in this study may be targeted for improvement in RubisCOs from other sources, including crop plants.

## Abstract (precis)

RubisCO catalysis has a significant impact on mitigating greenhouse gas accumulation and CO_2_ conversion to food, fuel, and other organic compounds required to sustain life. Because RubisCO-dependent CO_2_ fixation is severely compromised by oxygen inhibition and other physiological constraints, improving RubisCO’s kinetic properties to enhance growth in the presence of atmospheric O_2_ levels has been a longstanding goal. In this study, RubisCO variants with superior structure-functional properties were selected which resulted in enhanced growth of an autotrophic host organism (R. capsulatus), indicating that RubisCO function was indeed growth limiting. It is evident from these results that genetically engineered RubisCO with kinetically enhanced properties can positively impact growth rates in primary producers.

## INTRODUCTION

Urbanization, industrialization and technological advances impact anthropogenic contributions to increases in atmospheric CO_2_ levels and global warming. In the context of increasing environmental challenges and global demands for food and fuel, studying the basis of biological carbon sequestration and potentially improving the process is a reasonable goal. Nature’s choice of ribulose 1,5-bisphosphate carboxylase/oxygenase (RubisCO), the world’s most abundant enzyme, as the principal biocatalyst to convert atmospheric CO_2_ into usable organic carbon underpins continuing efforts to improve the enzyme’s performance (1–5). RubisCO is central to carbohydrate biosynthesis in photosynthetic eukaryotes and a diverse group of prokaryotes, including photo- or chemoautotrophic bacteria that inhabit aerobic, semiaerobic, or completely anoxic environments (1).

There are four structural forms of RubisCOs (1). Hexadecamers of eight large (∼55-kDa) and eight small (∼12- to 15-kDa) subunits (L_8_S_8_) make up the form I RubisCOs, which are present in plants, cyanobacteria, and most algae and autotrophic bacteria. Forms II, III, and IV are proteins that assemble as higher-order oligomers of a catalytic dimer of large subunits ([L_2_]*_n_*) and are present in bacteria and archaea. Functionally, form I, II, and III RubisCOs catalyze both ribulose 1,5-bisphosphate (RuBP) carboxylation and oxygenation but also have a distinct role in sulfur metabolism (6). However, the form IV RubisCO-like proteins are unable to fix CO_2_ but play major roles in sulfur salvage metabolism (1, 6). It is now possible to assemble a plant RubisCO in Escherichia coli, a model bacterium of choice (7). However, most of what we know about the enzymatic mechanism, diversity and evolution, structure-function relationships, and regulation has been inferred from studies with diverse prokaryotic RubisCOs (1, 8, 9).

The catalytic mechanism of RubisCO involves CO_2_ or O_2_ addition onto a 2,3-enediol intermediate derived from RuBP (8). The O_2_ addition initiates a photo-oxidative respiratory pathway, which consumes ATP and results in stoichiometric release of one molecule each of CO_2_ and NH_3_ per cycle. Although naturally occurring RubisCOs have evolved to favor CO_2_ addition, atmospheric concentrations of CO_2_ (∼0.04%) and O_2_ (∼21%) can result in losses of more than 50% of the fixed organic carbon in plants (10). The CO_2_/O_2_ substrate specificity factor (Ω) is a kinetic constant that denotes the ratio of carboxylation versus oxygenation efficiencies of RubisCO at any given concentrations of CO_2_ and O_2_. The Ω value and other structure-functional properties vary widely among divergent RubisCOs (9, 11–14). Although it is intuitive to use Ω as a yardstick for measuring and improving the CO_2_ fixation efficiency of RubisCO in an organism, evolutionary adaptations point to multiple mechanisms by which nature has optimized CO_2_ fixation in different environments. This includes RubisCO structure-functional improvements, CO_2_-concentrating mechanisms, and the utilization of C_4_ and crassulacean acid metabolism (CAM) pathways in plants (9, 15, 16). It is thus imperative to better understand the basis of the enzyme’s bifunctionality, its relationship to the evolution of divergent RubisCOs, and the associated cellular adaptations that contribute to optimized CO_2_ fixation (1, 17).

Heterologous or hybrid RubisCOs have been expressed in E. coli (18–21), autotrophic bacteria (*Synechococcus*, Rhodopseudomonas palustris, Rhodobacter capsulatus, and Ralstonia eutropha) (22–25), the green alga Chlamydomonas reinhardtii (26), and the plants Arabidopsis thaliana (27), rice (28), and tobacco (29–31). However, simple model organisms that are associated with facile growth requirements and established tools for genetic manipulation have long facilitated structure-function studies with divergent RubisCOs and provided fascinating insights into other auxiliary factors required for RubisCO function (1, 4, 9, 32, 33). Among these, the RubisCO deletion mutant of R. capsulatus (strain SB I/II^−^) has been used for both directed evolution and functional selection of previously uncharacterized RubisCOs encoded by genes present in environmental DNA samples (34–36). R. capsulatus is a metabolically versatile nonsulfur purple photosynthetic bacterium that can be cultured under photo- or chemoautotrophic conditions (i.e., CO_2_-dependent growth) or under heterotrophic conditions (i.e., with an externally supplemented organic carbon source) in the presence (chemotrophic) or absence (phototrophic) of oxygen.

The form I RubisCO from *Synechococcus* structurally resembles the plant enzyme but has a much higher *K_m_* for CO_2_ (*K_c_*, ∼180 μM) and a lower specificity factor (Ω = ∼40) relative to the values characteristic of a typical plant enzyme (e.g., for spinach RubisCO, *K_c_* = ∼20 μM and Ω = 80) (23, 37). The sequestration of *Synechococcus* RubisCO into CO_2_-concentrating carboxysomes *in vivo* presumably explains the lack of selective pressure to naturally evolve a “better” kinetic variant (12, 15, 23). The *Synechococcus* enzyme has thus been an excellent model enzyme for directed evolution in heterologous hosts that lack a carbon-concentrating mechanism (20, 23, 38, 39). Complementation of the R. capsulatus RubisCO deletion strain with *Synechococcus* sp. strain PCC 6301 RubisCO genes allowed the selection of several mutant substitutions that both positively and negatively influenced activity and interactions with CO_2_ or O_2_, resulting in the identification of a semiconserved hydrophobic region adjacent to the active site (23, 38, 39). Subsequent studies targeting equivalent residues in *R. eutropha* form I and archaeal Archaeoglobus fulgidus or Thermococcus kodakarensis form III RubisCOs resulted in the identification of mutants with beneficial changes to the enzymes’ oxygen sensitivity (25, 40, 41), leading to the conclusion that this hydrophobic region in divergent enzymes could be a critical contributor for differential interactions with CO_2_ and O_2_ during catalysis. In the current study, additional residues in this hydrophobic region of the *Synechococcus* form I RubisCO were analyzed using site-directed mutagenesis. In addition, random mutagenesis and suppressor selection with negative-mutant genes resulted in the identification of second-site suppressor mutations in the structural genes encoding both large and small subunits of the enzyme. Detailed structure-function analyses point to the importance of additional hydrophobic regions and the large-small subunit interface for differential interactions with CO_2_ and O_2_. Selection of mutant enzymes with enhanced catalytic properties that confer superior CO_2_-dependent growth phenotypes accentuates the utility of nonnative autotrophic host systems for artificial evolution of RubisCO variants with attendant physiological consequences.

## RESULTS

### Analysis of residues in hydrophobic regions of *Synechococcus* form I RubisCO.

**FIG 1:**
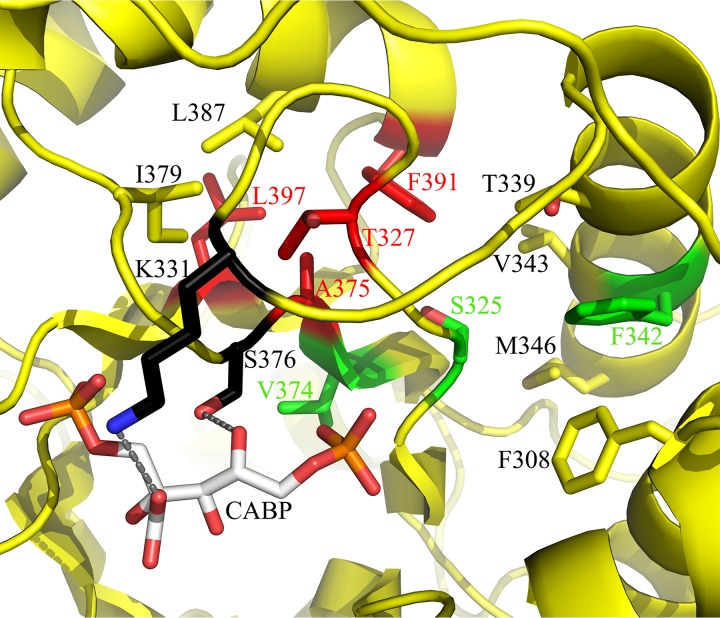
Hydrophobic region adjacent to the active site in the X-ray crystal structure of activated *Synechococcus* form I RubisCO (yellow; PDB ID 1RBL). Relevant residues are shown in stick representation and labeled. The transition state analog carboxyarabinitol-1,5-bisphosphate (CABP) is colored gray, and the active-site residues are colored black. Gray dotted lines represent van der Waals interactions between the active-site residues and CABP. Residues Ala^375^, Thr^327^, Phe^391^, and Leu^397^ (red) are within 4 Å of each other and were targeted for directed mutagenesis. Residues in this region that were identified via suppressor selection are colored green. For better clarity, the terminal atoms are colored based on electronegativities (oxygen, red; nitrogen, blue, phosphorus, orange; sulfur, yellow).

**FIG 2:**
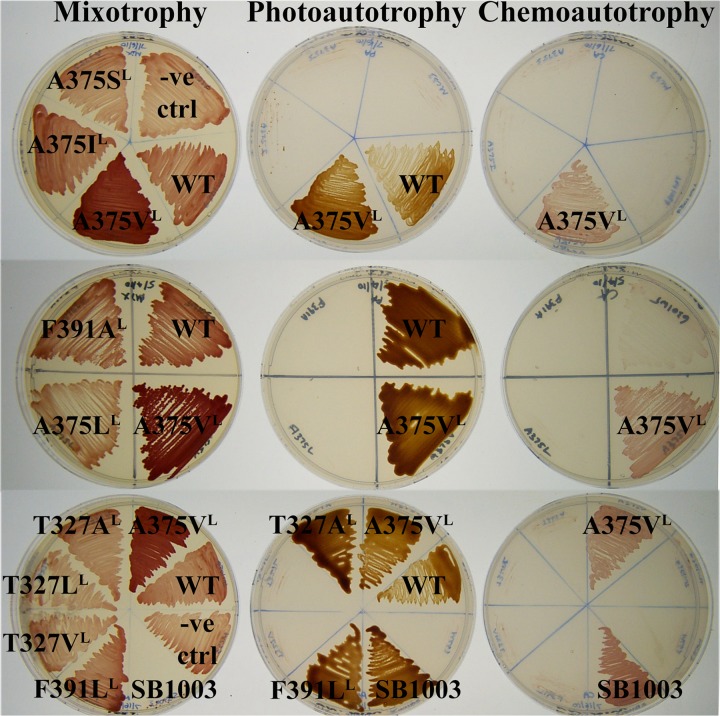
Growth phenotypes of R. capsulatus wild type (strain SB1003) and the RubisCO deletion mutant strains that had been complemented with wild type (WT) or site-directed mutants (labeled with respective residue substitutions) of *Synechococcus* RubisCO. Mixotrophic growth was assessed on rich (peptone-yeast extract) medium supplemented with tetracycline (to select for plasmid-complemented strains) and a gas mixture comprising 5% CO_2_ and 95% H_2_. CO_2_-dependent growth was assessed on minimal medium supplemented with a gas mixture comprising either 5% CO_2_ and 95% H_2_ gas mixture (photoautotrophy) or 5% CO_2_, 45% H_2_, and 50% air (chemoautotrophy). The RubisCO deletion mutant strain that had been complemented with an empty plasmid was used as a negative control (“-ve ctrl”).

Previous studies identified mutant substitutions in residues Phe^342^ and Ala^375^ (Phe^345^ and Ala^378^ in spinach RubisCO) that led to improved structure-function properties of the enzyme (23, 32, 39, 42, 43). These two residues are in a hydrophobic region near the active site, which shows a striking pattern of conservation among the three forms of RubisCO (Table 1; Fig. 1). Residues in this region can directly impact the movement of invariant catalytic residues Lys^331^ and Ser^376^ (Lys^334^ and Ser^379^ in spinach RubisCO) during catalysis, thus affecting substrate RuBP binding and CO_2_/O_2_ specificity (8). The identity of Ala^375^ in the *Synechococcus* form I RubisCO, or its equivalent in other RubisCOs, appears to be specifically important for differential interactions with CO_2_ and O_2_ (25, 39–41). Hence, other conserved and semiconserved nonpolar residues in van der Waals contact with Ala^375^ (within 4 Å) were targeted for mutagenic analysis (Table 1; Fig. 1). The invariant Thr^327^ was changed to an alanine (neutral) or a valine or a leucine (nonpolar), Phe^391^ was changed to an alanine or a leucine (to reflect its identity in other RubisCOs), and Leu^397^ was changed to an alanine. Ala^375^ was also changed to a leucine because substitution with a shorter (valine) or a bulkier (isoleucine) branched-chain hydrophobic residue resulted in contrasting CO_2_-dependent growth phenotypes of R. capsulatus SB I/II^−^ (39). Similarly to the wild type, mutants T327A^L^ and F391L^L^ (superscript L refers to a mutant substitution in the *rbcL* gene) both could support CO_2_-dependent autotrophic growth of the host strain under anoxic conditions but not in the presence of oxygen. None of the other site-directed mutants could complement for CO_2_-dependent growth (Fig. 2). These growth responses accentuate the importance of the indicated residues in this region for enzyme function *in vivo*.

**TABLE 1:** Structure-based alignment of amino acid residues in a hydrophobic region near the active site in all three forms (IA, IB, IC, II, and III) of bona fide RubisCOs

Form and species	Residue(s) at position^*a*^:							
	308	327	342	346	375	387	391	397
	*	* *	* **	*	* *	*	*	*
Form I								
*Synechococcus* PCC 6301 (IB)	F	**S** G**T** VV-G K	TLG**F** V	M	P**VA** S GGI	L	**F**	**L**
Allochromatium vinosum (IA)	F	TGTVV-G K	TLGWI	L	AVA S GGI	L	F	L
*Spinacia oleracea* (IB)	F	SGTVV-G K	TLGFV	L	PVA S GGI	L	F	L
Nicotiana tabacum (IB)	F	SGTVV-G K	TLGFV	L	PEA S GGI	L	F	L
Chlamydomonas reinhardtii (IB)	F	SGTVV-G K	TLGFV	M	PVA S GGI	L	F	L
*Galdieria partita* (IC)	F	AGTVV-G K	TRGFY	L	PVA S GGI	L	L	L
Ralstonia eutropha (IC)	F	TGTAV-G K	VQGYY	C	PVA S GGI	L	F	L
Form II								
Rhodospirillum rubrum	A	TGTMGFG K	--AIA	L	PII S GGM	F	L	L
Thiobacillus denitrificans	A	VGTMGYG K	--IIA	I	PII S GGM	F	L	M
Rhodopseudomonas palustris	A	TGTMGFG K	--AIA	I	PII S GGM	F	L	N
Form III								
Thermococcus kodakarensis	M	VGTAGAG K	VIQNA	L	PTS S GGL	V	L	I
Archaeoglobus fulgidus	M	IGTAGAG K	TVQNA	F	PVS S GGL	V	L	I
Pyrococcus horikoshii	M	TGTAV-G K	IKRIN	L	PVA S GGL	L	L	

a Active-site residues are underlined. Residues identified via suppressor selection or targeted for mutagenesis are shown in bold. All residues in hydrophobic region adjacent to the active site are marked with an asterisk. Positions of equivalent residues in the *Synechococcus* RubisCO large subunit are indicated.

### Selection and phenotypes of second-site suppressors of hydrophobic-pocket residue mutants.

**FIG 3:**
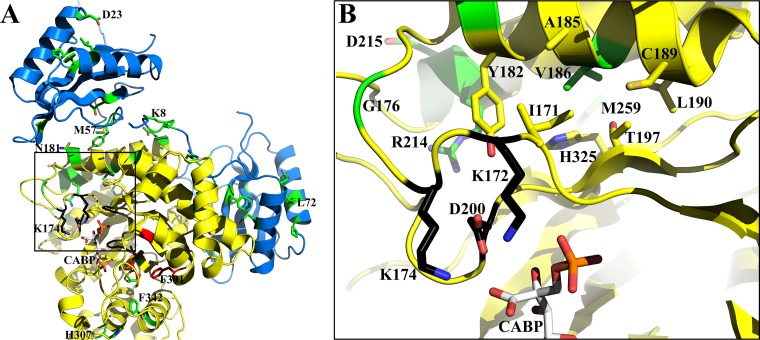
Distribution of residues identified via suppressor selection (green) in the holoenzyme structure of *Synechococcus* form I RubisCO (PDB ID 1RBL). (A) Residues that were targeted for mutagenesis (red) are shown in one of the large subunits (yellow). Two neighboring small subunits (blue) come in contact with a large subunit in the holoenzyme. Some residues are labeled to illustrate the position of the respective regions relative to the active site. (B) The boxed region in panel A showing interactions involving Val^186^ (green) is enlarged and shown with a few additional residues (yellow sticks) that are within 4 Å of the side chain of Val^186^. Active-site residues (black) in this region and the transition state analog CABP (light gray) are displayed in both panels. Terminal atoms in CABP and the amino acid side chains are colored based on electronegativities (oxygen, red; nitrogen, blue; phosphorus, orange; sulfur, yellow).

**FIG 4:**
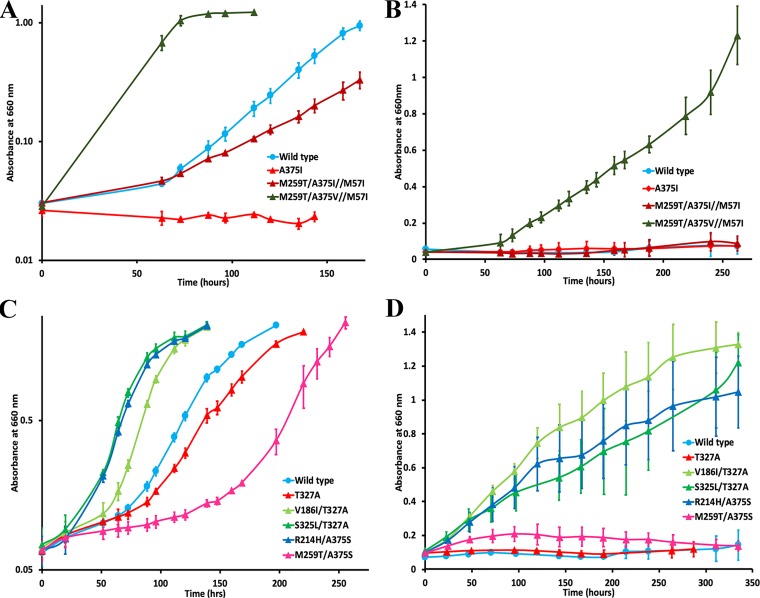
Growth responses of suppressor mutants in liquid cultures placed under anoxic (A and C) or oxic (B and D) CO_2_-dependent autotrophic growth conditions. Strain names indicate the mutant substitutions encoded by the large (*rbcL*) or small (*rbcS*) subunit genes of *Synechococcus* form I RubisCO. Large subunit mutant substitutions are separated by a shill, and the substitutions following a double shill are in the small subunit. Each curve was plotted with mean absorbance values measured from triplicate cultures, and the error bars represent the standard deviations for each data point. Data are representative of several independent growth experiments.

Mutant large subunit (*rbcL*) and wild-type small subunit (*rbcS*) genes of the negative mutants T327L^L^, T327V^L^, A375I^L^, A375L^L^, A375S^L^, F391A^L^, and L397A^L^ were used as the templates to generate a library of randomly mutagenized *rbcLS* genes in E. coli, conjugated *en masse* into R. capsulatus strain SB I/II^−^ and subjected to direct selection for CO_2_-dependent growth under anaerobic growth conditions. Multiple second-site suppressors were isolated for the negative mutant A375I^L^, and two second-site suppressors were isolated for each of the negative mutants A375S^L^ and A375L^L^ (Fig. 3; see also Table S1 in the supplemental material). A point mutation in mutant T327V^L^ led to the recovery of a pseudosuppressor (V327A^L^). No suppressors could be identified for the other negative mutants (i.e., T327L^L^, F391A^L^, and L397A^L^). For suppressor-mutant genes that had more than one point mutation in the same copy of the *rbcLS* gene cluster, individual point mutations were created with a template that carries the original mutation that caused the negative phenotype (Table S1) to determine if one of the individual mutations was sufficient for suppression. Whenever a suppressor mutant was identified, the resultant plasmid that had been reisolated from R. capsulatus and used for DNA sequencing was conjugated back into R. capsulatus strain SB I/II^−^ to verify that the phenotype was not an artifact of the selection procedure employed. In some cases, spontaneous mutations arose for negative mutants that were placed under selective growth conditions (i.e., with CO_2_ as the sole carbon source) for phenotype verification (Table S1). Spontaneous mutations were also identified with mutants that could support anoxic CO_2_-dependent growth but only when placed in liquid cultures under more stringent selective conditions (i.e., in the presence of oxygen). Three mutants were selected based on their ability to support oxygenic CO_2_-dependent growth of R. capsulatus strain SB I/II^−^. Two of them arose from additional mutations in the T327A^L^ mutant *rbcL* gene background, encoding either an S325L^L^ or a V186I^L^ substitution. Mutant M259T/A375V^L^//M57I^S^ (superscript “S” refers to a mutation in the *rbcS* gene; a single shill separates mutations occurring in the same large subunit; a double shill separates large and small subunit mutations) arose from mutant M259T/A375I^L^//M57I^S^, which had been selected under anoxic growth conditions (Table S1). Growth in liquid cultures provided a quantitative measure of the CO_2_-dependent growth responses conferred by the suppressors. Suppressors of mutant A375I^L^, which are able to complement for CO_2_-dependent growth only under anoxic conditions, all had growth rates that were generally lower than that of the wild type (Fig. S1). The R214H/A375S^L^ suppressor mutant, which was isolated under photoautotrophic (anoxic) growth conditions, and the three mutants selected under chemoautotrophic (oxic) growth conditions conferred better growth than the wild type under both anoxic and oxic conditions (Fig. 4). When created in isolation, V186I^L^, R214H^L^, and S325L^L^ single mutants were all able to complement for CO_2_-dependent growth under both anoxic and oxic conditions (Table S1). It is apparent that suppressor selection helped identify multiple regions in the quaternary structure of the enzyme (Fig. 3) that impact the enzyme’s interactions with CO_2_ and O_2_.

10.1128/mBio.01537-19.1 FIG S1 Liquid growth phenotypes of a representative set of suppressor mutants obtained for the A375I negative mutant. Mutant names point to the residue substitutions. The mutant A375V was used as a control, and its “positive” phenotype had been described previously (38). Data presented here are representative of multiple liquid growth experiments. All strains were grown in chemoheterotrophic medium to similar densities, and equal volumes of each culture were washed with minimal medium prior to inoculation in liquid minimal medium for autotrophic growth in cultures that were bubbled with a gas mixture containing 5% CO_2_/95% H_2_. Download FIG S1, TIF file, 0.1 MB. Copyright © 2019 Satagopan et al. 2019 Satagopan et al. This content is distributed under the terms of the Creative Commons Attribution 4.0 International license.

10.1128/mBio.01537-19.5 TABLE S1 Suppressor mutants isolated in this study, their derivatives, phenotypes, and the structural genes affected by them. Download Table S1, DOCX file, 0.02 MB. Copyright © 2019 Satagopan et al. 2019 Satagopan et al. This content is distributed under the terms of the Creative Commons Attribution 4.0 International license.

### Enzymatic properties of recombinant mutant and suppressor enzymes.

Net yields of recombinant enzymes with small subunit mutant substitutions were generally low. However, significant levels of soluble RubisCO could be purified from E. coli strains expressing mutant large subunit and wild-type small subunit genes. SDS-PAGE analysis of soluble and insoluble fractions of E. coli lysates indicated that abundantly synthesized subunits of the recombinant L397A^L^ mutant protein were present only in the latter. Supplementing the E. coli expression strain with chaperone DnaK, DnaJ, GrpE, GroEL, GroES, or Tf (TaKaRa) did not help with the assembly of L397A^L^ mutant RubisCO (data not shown). Among the site-directed mutants, T327A^L^ and F391L^L^ mutant substitutions resulted in enzymes that retained about 80% or 40% of the wild-type specific activity, respectively, but other recombinant mutant enzymes were devoid of activity (data not shown). The CO_2_-dependent growth complementation phenotypes of the site-directed mutants are consistent with the *in vitro* enzymatic activities (Fig. 2). All of the recombinant suppressor-mutant enzymes that could be purified retained lower levels of carboxylation specific activities than the wild type. Thus, the artificial selection procedures utilized in this study did not favor the isolation of mutant enzymes with enhanced carboxylation activity.

The ratio of carboxylase activities measured at limiting CO_2_ concentrations under 100% N_2_ versus 100% O_2_ (N_2_/O_2_ ratio) has been previously used to screen for RubisCO enzymes with altered kinetic properties (44). As part of the same assay, parallel determination of carboxylation activities in the presence of excess CO_2_, under 100% N_2_, provides a useful screen and measure of any changes to the enzyme’s *k*_cat_ value for carboxylation (44). Similar assays were performed with mutant *Synechococcus* RubisCOs. Several enzymes had lower N_2_/O_2_ ratios (Table S2), indicating that these enzymes were likely less inhibited in the presence of 100% O_2_. Further, the activity values measured under 100% N_2_ (i.e., absence of O_2_) with excess CO_2_ were reflective of the specific activity values obtained with purified enzymes. Enzymes with substantial levels of carboxylation activities, favorable N_2_/O_2_ ratios, and abilities to support better CO_2_-dependent growth than the wild type (i.e., M259T/A375V^L^//M57I^S^, R214H/A375S^L^, and V186I/T327A^L^) were chosen for further analysis of catalytic constants.

10.1128/mBio.01537-19.6 TABLE S2 Screen of RubisCO activities of recombinant wild-type and mutant *Synechococcus* form I RubisCOs measured at different CO_2_ and O_2_ levels. Download Table S2, DOCX file, 0.01 MB. Copyright © 2019 Satagopan et al. 2019 Satagopan et al. This content is distributed under the terms of the Creative Commons Attribution 4.0 International license.

Catalytic constants were determined with purified recombinant enzymes. The *K_m_* values for CO_2_ (*K_c_*) and O_2_ (*K_o_*) and the calculated *K_o_*/*K_c_* ratios were altered for most enzymes (Table 2), indicating that the residue changes indeed influence interactions with the two gaseous substrates. The significantly higher *K_o_*/*K_c_* ratios obtained for M259T/A375V^L^//M57I^S^ triple, V186I^L^ single, V186I/T327A^L^ double, S325^L^ single, and R214H/A375S^L^ double mutant enzymes likely account for the ability of these enzymes to support vigorous CO_2_-dependent growth under oxic conditions (Fig. 4). Notably, the *K_c_* values of M259T^L^ single, M259T/A375V^L^//M57I^S^ triple, V186I^L^ single, V186I/T327A^L^ double, S325^L^ single, and S325L/T327A^L^ double mutant enzymes were better than the wild-type value. The V186I/T327A^L^ double mutant enzyme also had a significantly higher *K_o_* value than the wild-type enzyme (Table 2). Despite the enzyme having a reduced *k*_cat_ value for carboxylation (49% lower than the wild-type value), the superior *K_o_*/*K_c_* ratio, unaltered values of Ω and *K*_RuBP_, and superior growth-complementation phenotypes (Fig. 4) indicate that the V186I/T327A^L^ suppressor mutant enzyme may be the best oxygen-tolerant RubisCO to have been artificially evolved thus far, conferring growth enhancement on an autotrophic host under physiologically relevant conditions.

**TABLE 2:** Kinetic properties of purified recombinant RubisCO enzymes^*a*^^,^^*b*^

Enzyme	Ω^*c*^ (*V_c_K_o_*/*V_o_K_c_*)	*k*_cat_ (s^−1^)	*K_c_*^*c*^ (μM CO_2_)	*K_o_*^*c*^ (μM O_2_)	*K_o_*/*K_c_*^*d*^	*K*_RuBP_^*c*^ (μM)
Wild type	41 ± 1	4.3 ± 0.7	190 ± 9	841 ± 30	4.4	29 ± 3
A375V^L^^*e*^	34	0.5	146	1,076	7.4	37
M259T^L^	42 ± 2	4.4 ± 1.0	147 ± 4	595 ± 13	4.0	30 ± 4
M259T/A375V^L^//M57I^S^	40 ± 3	2.2 ± 0.2	93 ± 11	716 ± 70	7.7	64 ± 7
R214H^L^	34 ± 3	3.6 ± 0.1	682 ± 30	2,152 ± 275	3.2	30 ± 4
R214H/A375S^L^	34 ± 1	0.6 ± 0.1	264 ± 22	1,731 ± 59	6.6	25 ± 5
T327A^L^	40 ± 2	2.8 ± 0.2	273 ± 19	1,303 ± 86	4.8	13 ± 2
V186I^L^	40 ± 2	2.8 ± 0.4	106 ± 5	686 ± 36	6.5	25 ± 2
V186I/T327A^L^	38 ± 1	2.1 ± 0.4	110 ± 14	1,189 ± 115	10.8	25 ± 2
S325L^L^	35 ± 1	4.0 ± 1.0	111 ± 14	931 ± 56	8.4	251 ± 17
S325L/T327A^L^	30 ± 1	2.6 ± 0.3	142 ± 23	577 ± 93	4.1	77 ± 8

a Enzymes that were identified or created based on suppressor selection from a common precursor (i.e., A375I^L^, A375S^L^, or T327A^L^) are placed into three separate groups. The A375I^L^ and A375S^L^ single-mutant enzymes had insignificant levels of RubisCO activity (39), and hence, the kinetic properties could not be determined for these enzymes.

b Kinetic constant values that favor better CO_2_ fixation rates (relative to wild type) are underlined.

c Values are the means ± standard deviation (*n* − 1) of at least three separate enzyme preparations.

d Calculated from measured *K_o_* and *K_c_* values.

e Values obtained from reference 39 and normalized with the wild-type values presented here.

### Effect of mutant substitutions on enzyme structure and assembly.

A significant number of *Synechococcus* form I RubisCO mutant enzymes previously isolated by artificial evolution were synthesized *in vivo* at higher levels or were more stable than the wild-type enzyme (32, 39, 43). To assess the soluble-protein levels of the selected suppressor mutant enzymes, R. capsulatus cells expressing the corresponding RubisCOs were harvested from photoautotrophically grown cultures, and soluble extracts were prepared using sonication and analyzed via SDS-PAGE and Western blotting. Several mutant enzymes with enhanced synthesis and stability were isolated in this study, including mutants R214H/A375S^L^ and M259T/A375I^L^//M57I^S^ (Fig. S2). With wild-type levels of RubisCO subunits, M259T/A375V^L^//M57I^S^ appears to have been preferentially selected for enhanced kinetic properties. No discernible trends were observed with specific activities measured from R. capsulatus cell extracts expressing various mutant proteins, although it could be concluded that RubisCO subunit synthesis and specific activities in cell extracts were significantly reduced in all strains that had been grown in the presence of O_2_ (data not shown).

10.1128/mBio.01537-19.2 FIG S2 SDS-PAGE (A and C) and Western blot (B and D) profiles of R. capsulatus soluble cell extracts expressing wild-type (lanes 2 and 12), mutant A375V^L^ (lane 13), and suppressor mutant (lanes 3 to 10 and 14 to 20) RubisCOs. The position of the RubisCO large subunit band (∼50 kDa) is indicated with an arrow. Sizes of proteins in the molecular weight standards (lanes 1 and 11) are indicated below each band in panel A. R. capsulatus cells used for this experiment were grown under anoxic photoautotrophic conditions. Samples electrophoresed correspond to lane numbers as follows: 3, V374A/A375I^L^; 4, V374A/A375V^L^; 5, A375I/A411T^L^; 6, A375I/E422Q^L^; **7, R214H/A375S^L^**; 8, M259T/A375S^L^; **9, S325L/T327A^L^**; **10, V186I/T327A^L^**; 13, A375V^L^; **14, M259T/A375I^L^//M57I^S^**; 15, A375I^L^//Q29R^S^; 16, A375I^L^//S16L^S^; 17, A375I^L^//L72F^S^; **18, M259T/A375V^L^//M57I^S^**; 19, G176S/A375I^L^; 20, A375L^L^//S16L^S^. Identical amounts of soluble protein (5 μg) were electrophoresed in all sample lanes. Mutants described in Fig. 4 (growth complementation phenotypes) and Table 2 (enzymatic properties) are highlighted in bold in this legend. Download FIG S2, TIF file, 0.8 MB. Copyright © 2019 Satagopan et al. 2019 Satagopan et al. This content is distributed under the terms of the Creative Commons Attribution 4.0 International license.

Because many of the kinetically altered suppressor mutant substitution enzymes appeared to be localized to the intersubunit interfaces (Fig. 3), their impact on the strength of large-large and large-small subunit interactions were assessed further using a bacterial two-hybrid system that had been previously utilized to show interactions between RubisCO subunits and regulator proteins (45). The levels of β-galactosidase provided a direct measure of the strength of large-small subunit interactions (Fig. S3). Several conclusions could be drawn from the two-hybrid analysis. The interaction strength of various large-small subunit pairs generally correlated with yields of recombinant enzymes that were purified from E. coli. For example, the interaction strengths of each of A375V^L^, A375I/A411T^L^, R214H/A375S^L^, V186I/T327A^L^, and S325L/T327A^L^ mutant large subunits with wild-type small subunit were better than what was measured with the wild-type large subunit (Fig. S3B and C). This correlated with consistently higher yields of the corresponding mutant RubisCOs in independent protein purification experiments (data not shown). Mutant substitutions appeared to selectively impact the large-small subunit interactions and not those between the large subunits themselves (Fig. S3A). The interaction strengths of a large-small subunit pair did not seem to be a determinant of the corresponding holoenzyme’s ability to support CO_2_-dependent growth of R. capsulatus SB I/II^−^ (Fig. S3B to D and Table S1). Last, substitutions in the small subunit appeared to generally diminish the strength of their interaction with the corresponding mutant large subunits, whereas the large subunit suppressor mutant substitutions appeared to generally improve the interaction strength with wild-type small subunits (Fig. S3C and D). In summary, the two-hybrid interaction strengths are reflective of the extent of subunit interactions that define protein assembly and turnover *in vivo*, particularly when the mutant proteins are expressed as recombinant enzymes in E. coli.

10.1128/mBio.01537-19.3 FIG S3 Bacterial two-hybrid reporter (β-galactosidase) assays with E. coli soluble cell extracts show various levels of interactions between the indicated protein pairs. The known interaction between the dimerization domain of the yeast transcriptional activator GAL4 (LGF2) and a domain derived from a mutant GAL11 protein (GAL11^P^) was used as a positive control (“Positive ctrl.”; dark green) (Stratagene). E. coli reporter cells cotransformed with the target and bait plasmids that had no inserts were used as a negative control (“Negative ctrl.”; white). Wild-type (WT) or mutant (represented by the respective mutant substitutions) large (L) and small (S) subunit genes that served as the target and bait sequences are labeled and separated by a double shill. Multiple mutant substitutions in the same large subunit sequence are separated by a single shill. (A) Two-hybrid interactions between large-small and large-large subunit pairs of wild-type (dark brown), mutant T327A^L^ (light brown), and negative mutant (red) enzymes. (B) Two-hybrid interactions between the large subunits of wild-type (dark brown) and Ala^375^ mutant enzymes that confer positive (green) or negative (red) phenotypes and wild-type small subunit. (C) Two-hybrid interactions of wild-type (dark brown), mutant T327A^L^ (light brown), negative mutant (red), or suppressor mutant (green) large subunits with wild-type small subunits. (D) Two-hybrid interactions of wild-type (dark brown), negative mutant (red), or suppressor mutant (green) large subunits with wild-type or mutant small subunits. With the exception of positive and negative controls, the activity values for all other combinations represent average values determined from at least 3 independent measurements. Error bars represent standard deviations. Download FIG S3, TIF file, 1.6 MB. Copyright © 2019 Satagopan et al. 2019 Satagopan et al. This content is distributed under the terms of the Creative Commons Attribution 4.0 International license.

Despite the enzyme having only ∼12% of the wild-type level of activity, the improved structural stability was an important determinant of the positive phenotype conferred by the A375V^L^ mutant enzyme (39). Thermal stability assays were performed to further assess the stabilities of recombinant enzymes. Whereas the M259T^L^ single, M259T/A375V^L^//M57I^S^ triple, and V186I/T327A^L^ double mutant enzymes lost only 2 to 14% activity after a 5-min incubation at 60°C, the wild-type enzyme lost 24% of its initial activity after 5 min (Fig. S4). However, after a 60-min incubation, only the M259T^L^ mutant enzyme retained higher levels of activity than the wild-type sample. Thus, although several suppressor mutant substitutions appear to enhance structural interactions, all of them do not confer physiologically significant phenotypes.

10.1128/mBio.01537-19.4 FIG S4 Thermal stabilities of suppressor mutant *Synechococcus* RubisCOs. Aliquots of each recombinant enzyme were incubated at 60°C, and at various times, RuBP carboxylation activities were measured at 25°C in standard assays. For each enzyme, the activities retained after the respective incubation times were plotted as a percentage of the activity in the untreated sample. Data shown here are from a single experiment, but the thermal stability profile of the wild-type enzyme is consistent with results from other independent experiments performed with a different set of mutant enzymes. Download FIG S4, TIF file, 0.4 MB. Copyright © 2019 Satagopan et al. 2019 Satagopan et al. This content is distributed under the terms of the Creative Commons Attribution 4.0 International license.

## DISCUSSION

In this study, directed evolution resulted in the isolation and selection of cyanobacterial form I RubisCO mutant proteins with kinetic alterations that enhance CO_2_-dependent growth. The ability to artificially evolve enzymes that improve growth, specifically in the presence of O_2_, such as V186I/T327A^L^ and M259T/A375V^L^//M57I^S^, provides direct evidence that RubisCO can be functionally improved to play a physiologically significant role. In addition to positive kinetic variants, the selection procedures described here also allowed for the isolation of proteins with improved structural integrity. Structure-functional divergence and enhanced knowledge of the promiscuity of the RubisCO family of proteins have provided a better understanding of nature’s constraints governing the evolution of physiologically relevant enzymatic properties such as oxygen tolerance. Several studies have highlighted the importance of molecular chaperones and other accessory proteins for gene expression (46), functional assembly, activity regulation, and evolvability of divergent RubisCO molecules in heterologous hosts (4, 9). Despite the constraints placed by these requirements, heterologous RubisCO genes have been successfully expressed and the resultant proteins functionally assembled in hosts like E. coli, R. capsulatus, R. palustris, and *R. eutropha*, utilizing only the native host cell’s regulatory and assembly machineries (9, 20). When the wild-type *Synechococcus rbcLS* genes are expressed in E. coli or R. capsulatus, the amounts of RubisCO subunits in the soluble fractions are normally small, resulting in the selection and isolation of several suppressor mutants with substitutions that confer higher levels of RubisCO protein in the soluble fraction (see Fig. S3 in the supplemental material) (32). However, the selection of suppressors V186I/T327A^L^ and S325L/T327A^L^ from mutant T327A^L^ and R214H/A375S^L^ from mutant A375S^L^ and the sequential evolution of mutant M259T/A375V^L^//M57I^S^ from mutant A375I^L^ is direct evidence that physiologically significant growth enhancements are achievable via primary changes to the functional properties of the enzyme, specifically the altered interactions of these enzymes with substrates CO_2_ and/or O_2_.

### Utility of RubisCO bioselection systems with autotrophic growth capabilities.

Autotrophic bacteria such as R. capsulatus, R. palustris, and *R. eutropha* have been exploited for selection studies with heterologous RubisCOs (23–25, 39, 47, 48). The absolute dependence on RubisCO for CO_2_-dependent growth, coupled with the ability to grow under heterotrophic growth conditions (i.e., RubisCO and the Calvin-Benson-Bassham [CBB] cycle are dispensable), allows for a convenient means to select suppressor mutations in RubisCO genes that overcome an initial negative-growth phenotype. In this study, we identified and selected multiple mutants of *Synechococcus* sp. PCC 6301 form I RubisCO with the R. capsulatus host strain cultured at various levels of stringency, thereby identifying structural regions of the enzyme (i.e., bulky nonpolar side chains in hydrophobic regions) that were altered to enhance function (i.e., *K_c_* or *K_o_* values) and support CO_2_-dependent growth. Convergent identification of several residues in the *Synechococcus* enzyme using both R. capsulatus (Table S1) and E. coli RubisCO selection systems (20, 32, 49) indicates that the regions surrounding these residues may be the most readily accessible hot spots for targeted improvements in RubisCO’s properties under aerobic conditions.

### The importance of hydrophobic regions and the large-small subunit interface in form I RubisCOs.

In a previous study, molecular dynamics (MD) simulations were performed to investigate the movement of CO_2_ and O_2_ in and around RubisCO. It was proposed that all form I RubisCOs are able to preferentially sequester CO_2_ in hydrophobic regions that are continuous and connect to the active site (50). These simulations also pointed out that the small subunits may act as CO_2_ reservoirs. If this is true, the mutant substitutions identified in this study may represent the various CO_2_-sequestering regions. It is thus reasonable to expect these regions to impact net CO_2_ availability in the vicinity of the active site.

Residues Phe^342^ and Ala^375^ have been identified via selection in this and previous studies (20, 23, 32, 42, 43, 49). Substitution of Phe^342^ or the equivalent residue in other RubisCOs mostly affected the enzyme’s structural stability or the *K*_RuBP_ (20, 25, 32, 49). However, substitution of Ala^375^ in the *Synechococcus* form I enzyme or its equivalent in other enzymes resulted in beneficial changes to the enzymes’ *K_c_* and *K_o_* values (25, 39, 41). Suppressor selection in *R. eutropha* with an A380V^L^ negative mutant identified T330A^L^ or Y348C^L^ as a second-site suppressor (25). These amino acid residues (Ser^325^ and Val^343^ in *Synechococcus* RubisCO) are part of the same hydrophobic region in which the T327A^L^ and S325L^L^ mutant substitutions are present (Fig. 1). It was thus not surprising that the presence of an S325L^L^ mutant substitution complements the T327A^L^ single mutant enzyme for better CO_2_ fixation under oxic conditions.

**FIG 5:**
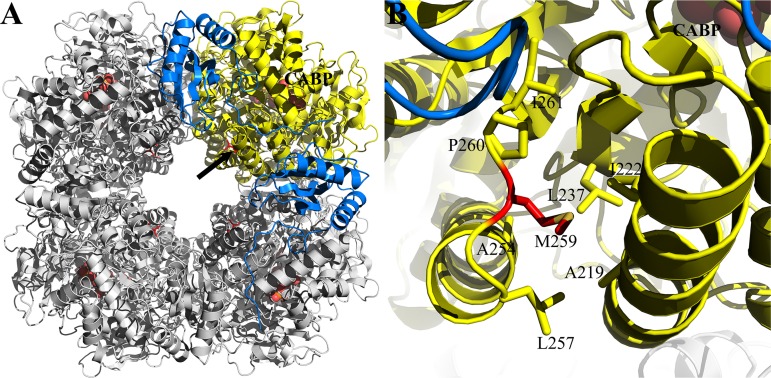
Position of residue Met^259^ in the X-ray crystal structure of *Synechococcus* form I RubisCO (PDB ID 1RBL). (A) The side chain of Met^259^ (red sticks) is at the interface of large (yellow/gray) and small (blue/gray) subunits, lining the central solvent channel in the holoenzyme. Its position in one of the large subunits (yellow) is indicated with an arrow. The position of CABP, the transition state analog (shown as spheres), which is present in all eight active sites, is labeled in one of them (yellow). (B) A closeup view of the region surrounding Met^259^, showing hydrophobic interactions (within 4 Å) with other nonpolar residues (yellow; stick representation).

Two independently selected suppressor mutants (M259T/A375I^L^//M57I^S^ and M259T/A375S^L^) both had an M259T^L^ mutation. M259T^L^ has also been selected in previous studies (23, 32, 43, 49). The side chain of Met^259^ is in a second hydrophobic region that is adjacent to the central solvent channel and the large-small subunit interface (Fig. 5). Although far away from the active site, the M259T^L^ mutant substitution confers structural stability and an improved *K_c_* value, consistent with what has been reported before (32, 43). These changes account for the improved CO_2_-dependent growth phenotype conferred by M259T^L^. Suppressor selection and targeted modification of large and small subunit residues in this region led to the creation of a *Chlamydomonas* RubisCO variant with enhanced kinetic properties resembling a plant enzyme (37, 51–53, 58). This provided a rationale for understanding how the M259T^L^ and M57I^S^ substitutions could complement to restore function in the A375I^L^ negative mutant and further lead to the accumulation of an IA375V^L^ pseudosuppressor, resulting in a positive phenotype. Other small subunit suppressors were isolated for the Ala^375^ mutants, but they resulted in either reduced recombinant protein yields or low specific activities, precluding them from further analysis.

Val^186^ is a conserved residue in a third hydrophobic region that connects the large-small subunit interface near Met^259^ to the other side of the active site via Lys^172^ (Lys^175^ in spinach RubisCO) (Fig. 3B). A V186I^L^ single mutant was identified and analyzed in a previous study (20), and its enzymatic properties are generally consistent with what is reported here (Table 2). However, the isolation of V186I^L^ as a second-site suppressor of T327A^L^ in this study indicates that the two distal hydrophobic regions (Fig. 1 and 3) are likely connected via their coordinated interactions with incoming CO_2_. Val^186^ is surrounded by other conserved residues that have also been identified via suppressor selection in this study (Fig. 3B). Whereas a G176S^L^ substitution was isolated as a suppressor for the A375I^L^ negative-mutant substitution (Table S1), a previous bioselection screen identified G176D^L^ as a negative mutant with altered CO_2_ interactions (38). Substitution of the conserved Cys^192^ in the *Chlamydomonas* form I RubisCO (Cys^189^ in the form I *Synechococcus* RubisCO) resulted in an enzyme with an altered *K_o_*/*K_c_* ratio (54).

Although several residues that were targeted or identified in this study have been independently identified and analyzed in previous investigations, the unique physiological context provided by the R. capsulatus system underscores the functional significance of complementing structural interactions. This study has also resulted in the concerted identification of other conserved residues in the large-large (Phe^37^, Ala^53^, Lys^249^, and His^307^) and the large-small subunit interfaces (residues Asn^181^, Gly^192^, Arg^214^, Glu^228^, Ala^411^, and Glu^422^ in the large subunit and residues Ser^16^, Gln^29^, Glu^43^, Tyr^54^, Met^57^, and Leu^72^ in the small subunit). Suppressor-mutant combinations involving these residues provide new insights regarding complementary structural interactions that may be targeted for evolving RubisCO variants with more predictable structure-function properties.

In conclusion, the R. capsulatus selection strategy has been successfully employed to evolve *Synechococcus* RubisCO variants with selective improvements in the enzyme’s interactions with CO_2_ versus O_2_. These results bode well for performing directed evolution and selection studies with other RubisCOs that may be functionally expressed in R. capsulatus. Because prokaryotic RubisCOs function under diverse metabolic contexts (1), it should be possible to learn more about the enzyme’s structure-function relationships by tapping into diverse microbial genomes for potentially “evolvable” RubisCO genes. As previously indicated (34, 35), the vastly uncultured “microbial dark matter” could be yet another treasure trove for identifying structurally diverse RubisCO genes. Conserved identities of residues identified in this study will facilitate targeted approaches to improve RubisCO’s performance in other divergent organisms that contribute significantly to global CO_2_ fixation.

## MATERIALS AND METHODS

### Bacterial strains, culture conditions, and plasmids.

Strains SB1003 and SB I/II^−^ are the wild-type and RubisCO deletion strains, respectively, of Rhodobacter capsulatus (25). R. capsulatus was cultured aerobically under chemoheterotrophic or chemoautotrophic (CO_2_-dependent) growth conditions and anaerobically under photoautotrophic (CO_2_-dependent) growth conditions at 30°C, as described previously (39). Top10 (Thermo Fisher) or XL1-Blue MRF′(Agilent) strains of E. coli were used for cloning procedures. Strain S17-1 (ATCC 47055) was used for mobilizing plasmids into R. capsulatus strain SB I/II^−^ (47). Strain FW102 (55), which contains the *lac* operon under the control of the λ operator, was used as a reporter strain for bacterial two-hybrid assays. Strain BL21(DE3) was used for recombinant protein synthesis. In some cases, this strain was supplemented with chaperone plasmids (TaKaRa) to facilitate assembly of poorly soluble proteins. E. coli cells were cultured aerobically in lysogeny broth (LB) at 37°C with shaking at 250 rpm. For protein purifications, cells were grown, induced, and harvested as described previously (39). A pUC19 clone with *Synechococcus rbcLS* genes (23) was used as a template for site-directed mutagenesis. A broad-host-range plasmid, pRPS-MCS3, was used for complementation studies with R. capsulatus strain SB I/II^−^ (39). Plasmids pET28a and pET11a (Novagen) were used for gene expression in E. coli.

### Mutagenesis, molecular biology procedures, matings, and selection.

Site-directed mutagenesis was performed using a QuikChange kit (Agilent). Random mutagenesis of *Synechococcus rbcLS* genes was accomplished using either error-prone PCR amplification (39, 47) or chemical mutagenesis using *N*-methyl-*N*′-nitro-*N*-nitrosoguanidine (MNNG) as a mutagen and a previously described procedure (59) that was modified as described here. E. coli cells with template plasmids were grown to an optical density (OD) of 0.7 at 600 nm; washed and resuspended in 0.1 M citrate buffer, pH 5.5, with MNNG added to a final concentration of 200 μg ml^−1^; and incubated at 30°C for 30 or 60 min. Cells were then washed in 0.1 M phosphate buffer, pH 7.0, and grown for 1 h in selective LB medium prior to conjugation. Nonmutagenic PCRs were performed with PrimeSTAR GXL DNA polymerase (Clontech). Cloning procedures utilized restriction enzymes and T4 DNA ligase purchased from Thermo Fisher Scientific or New England Biolabs. DNA sequences were verified by Sanger DNA sequencing (Plant-Microbe Genomics Facility, The Ohio State University).

Plasmids were mobilized from E. coli into R. capsulatus using either a triparental or diparental mating strategy (39, 47). After mating, the recipient R. capsulatus host cells were selected on either chemoheterotrophic (antibiotic selection) or autotrophic (CO_2_-dependent growth selection) media (39, 47). Natural selection for spontaneous mutations occurred in liquid or solid autotrophic media with R. capsulatus strains containing negative-mutant (no activity supported) RubisCO genes.

### Bacterial two-hybrid assays.

The BacterioMatch II two-hybrid system (Agilent) was used to compare interaction strengths between wild-type and mutant RubisCO subunits. The genes encoding the large (*rbcL*) and small (*rbcS*) subunits were cloned into the pTRG (target) and pBT (bait) plasmids, respectively. Reporter assays were carried out as described previously (45).

### Preparation of cell extracts, purification of RubisCO, and biochemical and structure analysis.

Autotrophically grown R. capsulatus liquid cultures were harvested after reaching the stationary phase (OD, ∼1.2 to 1.5 at 660 nm) by centrifugation at 8,000 × *g* for 10 min at 25°C and washed and sonicated in Bicine buffer (50 mM Bicine-NaOH, 10 mM MgCl_2_, 10 mM NaHCO_3_, 1 mM dithiothreitol [DTT], pH 8.0). Supernatants were obtained by centrifugation at 16,100 × *g* for 10 min at 4°C and used for RubisCO-specific activity measurements, SDS-PAGE, and Western blot analyses (38, 39). Identical amounts of soluble protein (5 μg) were used in SDS-PAGE and Western blot analyses. RubisCO enzymes were purified as recombinant proteins with or without an N-terminal hexahistidine tag using plasmids pET11a and pET28a, respectively, using a two-step (39) or a three-step (47) procedure. Proteins were dialyzed into a Bicine buffer (50 mM Bicine-NaOH, 10 mM MgCl_2_, 1 mM DTT, 10 mM NaHCO_3_), concentrated using Amicon filters (MilliporeSigma), mixed with 20% glycerol, and stored as aliquots at −80°C. Protein concentrations were determined using the Bradford method using a dye reagent (Bio-Rad). All biochemicals were purchased from Sigma-Aldrich. PyMOL was used for protein structural analysis.

### Enzyme assays and determination of kinetic constants.

RuBP carboxylase activities were measured using radiometric assays that utilized NaH^14^CO_3_ (56) (Perkin Elmer). For the initial characterization, assays were performed with excess or limiting amounts of CO_2_ in vials flushed with 100% N_2_ or 100% O_2_ (44). Thermal stabilities of purified recombinant enzymes were determined by incubating aliquots of each enzyme (5 U) at 60°C for various times, cooling on ice, and determining remaining RuBP carboxylation activities at 25°C. Activity values were normalized against the activity in the untreated samples. Substrate specificity (Ω) values were determined with purified enzymes (50 μg) in assays performed under saturating (1.23 mM) oxygen concentrations (23). The [1-^3^H]RuBP that was required for these assays was synthesized and purified using standard methods (57). Kinetic constants *k*_cat_, *K_c_*, *K_o_*, and *K*_RuBP_ were determined using procedures described elsewhere (39, 47).
