# Supplementary figures and images for: Selection of Cyanobacterial (Synechococcus sp. Strain PCC 6301) RubisCO Variants with Improved Functional Properties That Confer Enhanced CO2-Dependent Growth of Rhodobacter capsulatus, a Photosynthetic Bacterium
Source: mBio. 2019 Jul 23;10(4):e01537-19. doi: 10.1128/mBio.01537-19 (PMC6650557; doi:10.1128/mBio.01537-19)

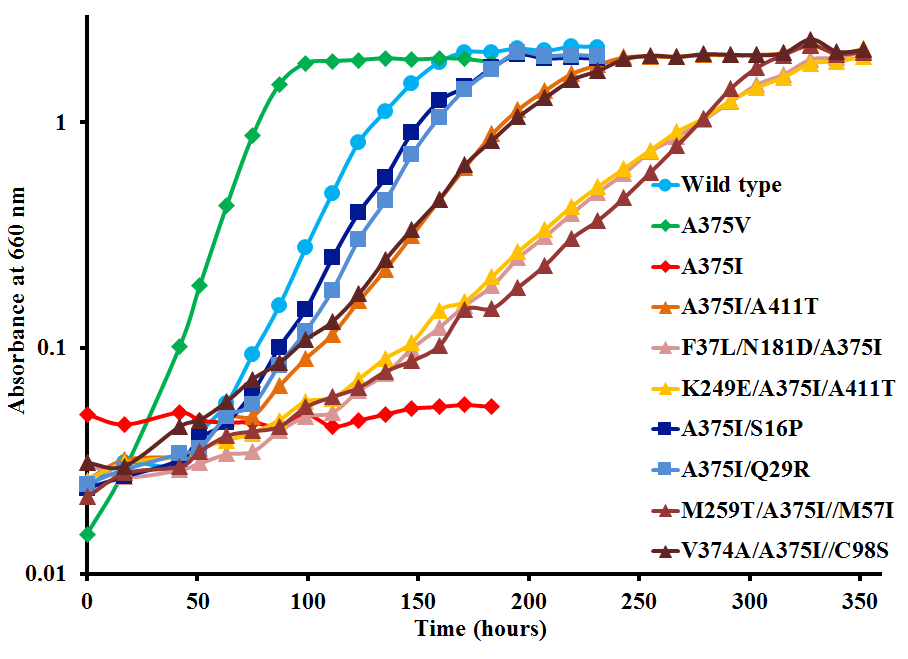

Supplement: FIG S1 [file mBio.01537-19-sf001.tif]

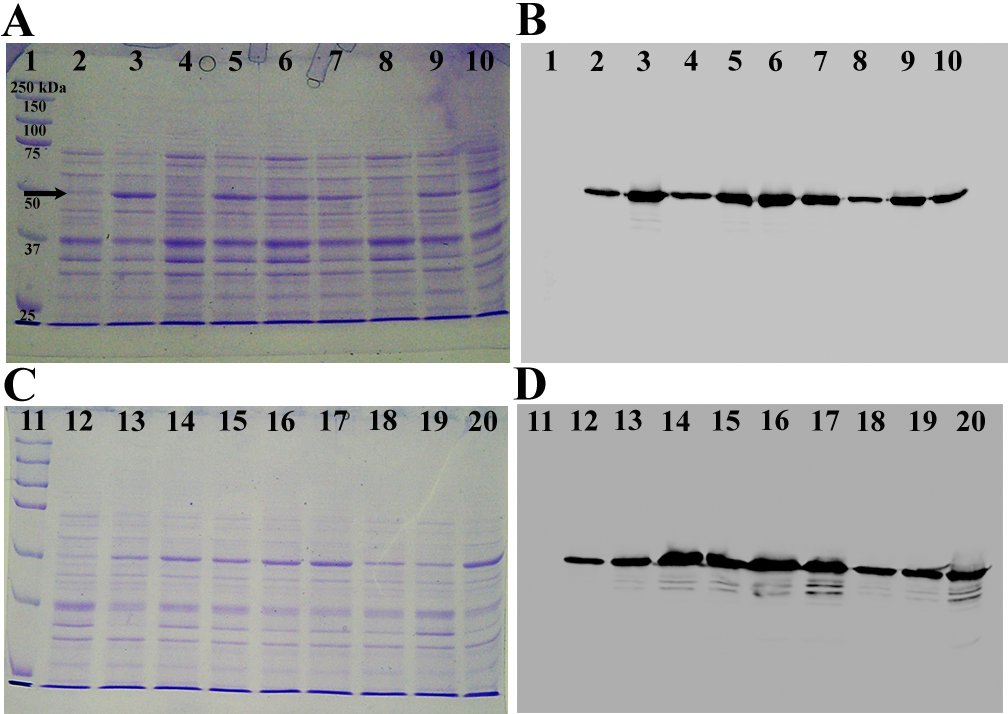

Supplement: FIG S2 [file mBio.01537-19-sf002.tif]

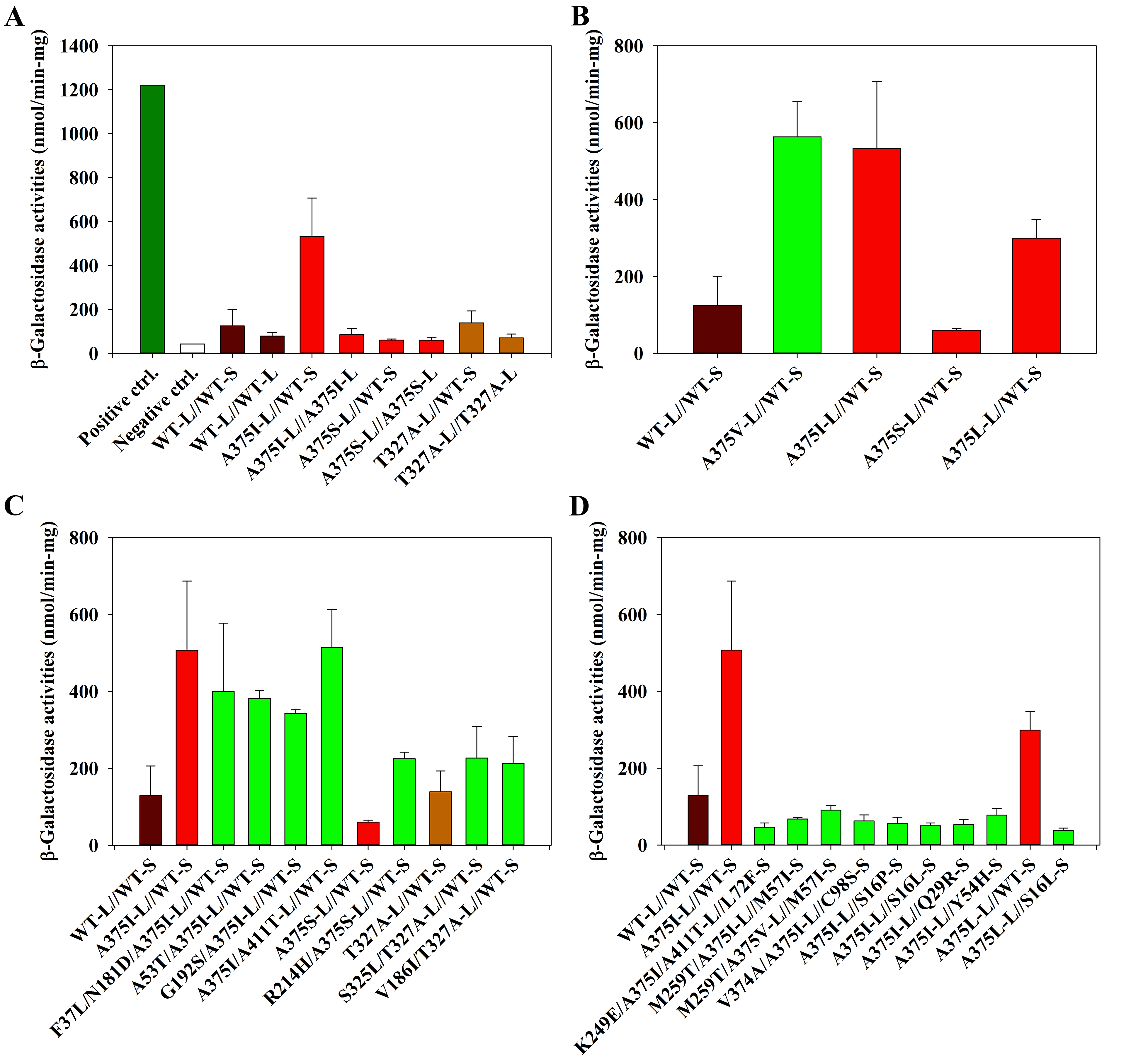

Supplement: FIG S3 [file mBio.01537-19-sf003.tif]

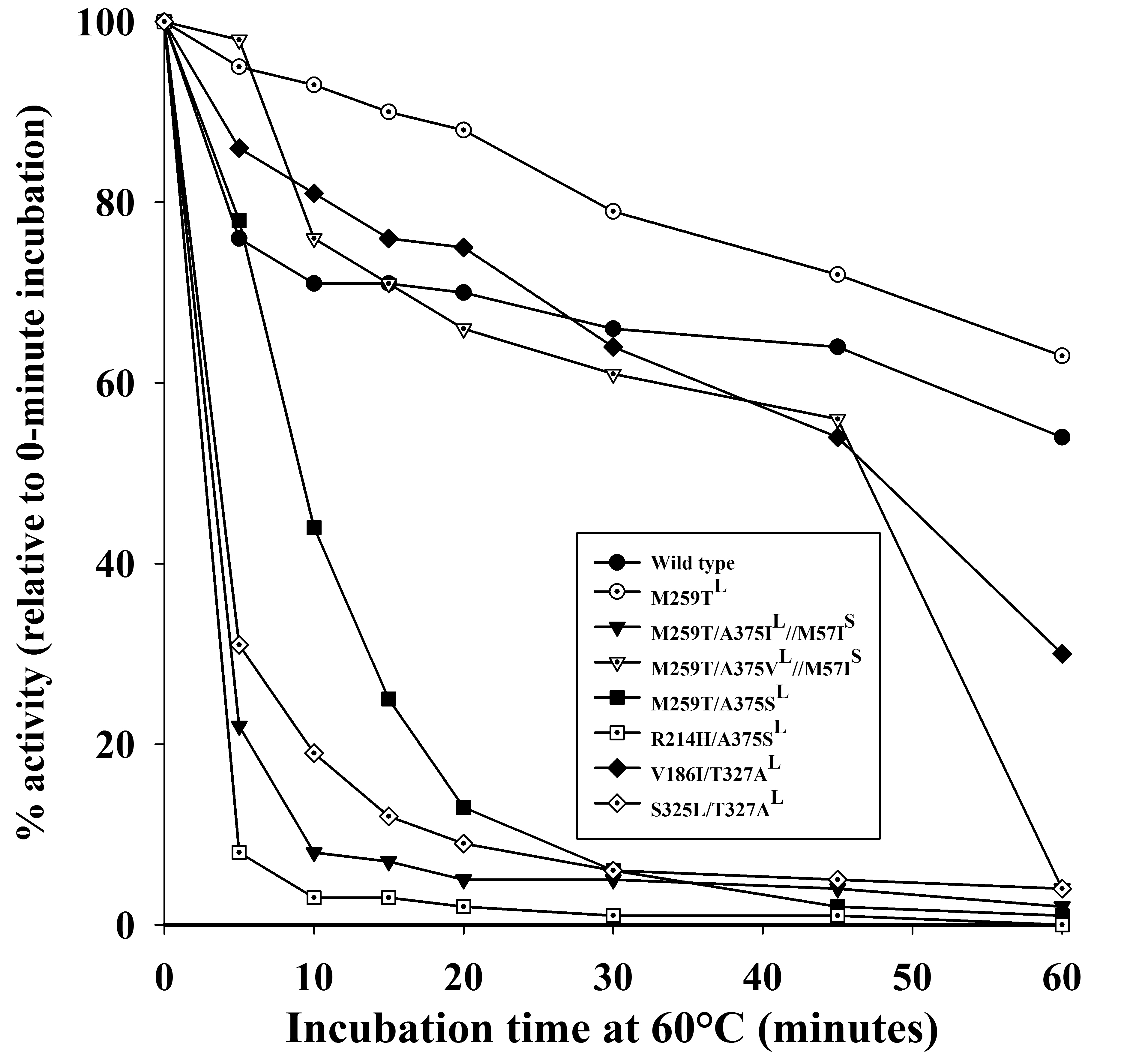

Supplement: FIG S4 [file mBio.01537-19-sf004.tif]
