# Supplementary material for: Selection of Cyanobacterial (Synechococcus sp. Strain PCC 6301) RubisCO Variants with Improved Functional Properties That Confer Enhanced CO2-Dependent Growth of Rhodobacter capsulatus, a Photosynthetic Bacterium
Source: mBio. 2019 Jul 23;10(4):e01537-19. doi: 10.1128/mBio.01537-19 (PMC6650557; doi:10.1128/mBio.01537-19)
Supplement: TABLE S1 [file mBio.01537-19-st001.docx]

**Table S1.** Suppressor mutants isolated in this study, their derivatives, phenotypes, and the structural genes affected by them.

| **Mutant ^a, b, c, d^** | **Photoautotrophic growth complementation** | **Chemoautotrophic growth complementation** | **Structural genes affected by mutations** | **Additional suppressors (spontaneous)** |
| --- | --- | --- | --- | --- |
| **A375I^L^** | **-** | **-** | **L** |  |
| A375I^L^/E422Q^L^ | + | - | L |  |
| G192S/A375I^L^ | + | - | L |  |
| G192S^L^ | - | - | L |  |
| A375I/A411V^L^ | + | - | L |  |
| A53T/A375I^L^ | + | - | L |  |
| G341V/A375I^L^ | + | - | L |  |
| G176S/A375I^L^ | + | - | L |  |
| G192D/A375I^L^ | + | - | L |  |
| E228K/A375I^L^ | + | - | L |  |
| F37L/N181D/A375I^L^ | + | - | L |  |
| F37L^L^ | - | - | L |  |
| N181D^L^ | + | - | L |  |
| F37L/A375I^L^ | - | - | L | M74I^L^, E43D^S^, Y54H^S^, L72F^S^ |
| N181D/A375I^L^ | - | - | L | M74I^L^, R212P/D213A^L^ |
| K249E/A375I/A411T^L^ | + | - | L |  |
| K249E^L^ | + | - | L |  |
| A411T^L^ | + | - | L |  |
| K249E/A375I^L^ | - | - | L | H307N^L^, G192C^L^, R11G^S^, R11L^S^ |
| A375I/A411T^L^ | + | - | L |  |
| A375I^L^//R11G^S^ | + | - | L, S |  |
| A375I^L^//S16P^S^ | + | +/- | L, S |  |
| S16P^S^ | + | - | S |  |
| A375I^L^//S16L^S^ | + | - | L, S |  |
| A375I^L^//D23K^S^ | + | - | L, S |  |
| A375I^L^//Q29R^S^ | + | +/- | L, S |  |
| Q29R^S^ | + | - | S |  |
| A375I^L^//Y54H^S^ | + | - | L, S |  |
| M259T/A375I^L^//M57I^S^ | + | - | L, S | A375V (oxic growth) |
| M259T^L^ | + | + | L |  |
| M57I^S^ | + | - | S |  |
| M259T/A375I^L^ | - | - | L |  |
| A375I^L^//M57I^S^ | - | - | L, S | R11C^S^, R11P^S^, S48L^S^. F103L^S^ |
| A375I^L^//P68L^S^ | + | - | L, S |  |
| A375I^L^//L72F^S^ | + | - | L, S |  |
| A375I^L^//P7S/L72F^S^ | + | - | L, S |  |
| V374A/A375I^L^//C98S^S^ | + | - | L, S |  |
| V374A^L^ | - | - | L | A375V^L^, F342L^L^ |
| C98S^S^ | - | - | S |  |
| V374A/A375I^L^ | + | - | L |  |
| A375I^L^//C98S^S^ | - | - | L, S | K8E^S^ |
| **A375L^L^** | **-** | **-** | **L** |  |
| A375L^L^//P68L^S^ | + | - | L, S |  |
| A375L^L^//S16L^S^ | + | - | L, S |  |
| **A375S^L^** | **-** | **-** | **L** |  |
| R214H/A375S^L^ | + | + | L |  |
| R214H^L^ | + | + | L |  |
| M259T/A375S^L^ | + | - | L |  |
| M259T^L^ | + | + | L |  |
| **T327A^L^** | + | - | L |  |
| V186I/T327A^L^ | + | + | L |  |
| V186I^L^ | + | + | L |  |
| S325L/T327A^L^ | + | + | L |  |
| S325L^L^ | + | + | L |  |

^a^ Site-directed mutants that were used as starting templates are shown in bold

^b^ Residues that were substituted in two or more suppressor mutants are highlighted by underlining the respective mutant substitutions

^c^ Mutants that were generated by site-directed mutagenesis or by combining previously identified mutations are right-justified

^d^ Mutant substitutions in the same subunit are separated by a “/” and those in different subunits are separated by a “//”. Superscripts ^“L”^ and ^“S”^ refer to mutations in *rbcL* and *rbcS*, respectively.
