# Supplementary material for: Selection of Cyanobacterial (Synechococcus sp. Strain PCC 6301) RubisCO Variants with Improved Functional Properties That Confer Enhanced CO2-Dependent Growth of Rhodobacter capsulatus, a Photosynthetic Bacterium
Source: mBio. 2019 Jul 23;10(4):e01537-19. doi: 10.1128/mBio.01537-19 (PMC6650557; doi:10.1128/mBio.01537-19)
Supplement: TABLE S2 [file mBio.01537-19-st002.docx]

**Table S2.** Screen of RubisCO activities of recombinant wild-type and mutant *Synechococcus* form I RubisCOs measured at different CO_2_ and O_2_ levels

| **Enzyme** | **Carboxylase activities at 25°C^a^**  **(nmol/min-mg protein)** | | | **N_2_/O_2_ ratio ^b^**  **(A/B)** |
| --- | --- | --- | --- | --- |
|  | **100% N_2_** | | **100% O_2_** |  |
|  | **High CO_2_**  **(649 μM CO_2_)** | **Low CO_2_**  **(59 μM CO_2_)** | **Low CO_2_**  **(59 μM CO_2_)** |  |
|  |  | **(A)** | **(B)** |  |
| Wild type | 2071 | 358 | 189 | 1.9 |
| M259T^L^ | 3557 | 992 | 492 | 2.0 |
| A375V^L^ | 308 | 130 | 98 | 1.3 |
| M259T/A375I^L^//M57I^S^ | 38 | 40 | 17 | 2.4 |
| M259T/A375V^L^//M57I^S^ | 775 | 324 | 213 | 1.5 |
| R214H/A375S^L^ | 238 | 34 | 25 | 1.4 |
| S325L/T327A^L^ | 912 | 266 | 123 | 2.2 |

*^a^* Data obtained from a single experiment with a representative set of enzymes. Independent assays were performed with other mutant enzymes and comparable values were measured with overlapping control-enzymes (e.g., wild type, A375V^L^) between different experiments

*^b^* Calculated from experimentally determined activities (in two preceding columns)
